# Parenting around child snacking: development of a theoretically-guided, empirically informed conceptual model

**DOI:** 10.1186/s12966-015-0268-3

**Published:** 2015-09-17

**Authors:** Kirsten K. Davison, Christine E. Blake, Rachel E. Blaine, Nicholas A. Younginer, Alexandria Orloski, Heather A. Hamtil, Claudia Ganter, Yasmeen P. Bruton, Amber E Vaughn, Jennifer O. Fisher

**Affiliations:** Department of Nutrition, Harvard T.H. Chan School of Public Health, 665 Huntington Ave, Boston, MA 02445 USA; Department of Social and Behavioral Sciences, Harvard T.H. Chan School of Public Health, 665 Huntington Ave, Boston, MA 02445 USA; Department of Family and Consumer Sciences, California State University, Long Beach, , 1250 Bellflower Blvd, Long Beach, CA 90840-0501 USA; Department of Health Promotion, Education and Behavior, University of South Carolina, 915 Greene Street, Columbia, SC 29208 USA; Center for Obesity Research and Education, Temple University, 3323 N Broad St, Suite 175, Philadelphia, PA 19140 USA; Center for Health Promotion and Disease Prevention, University of North Carolina at Chapel Hill, 1700 Martin L. King Jr. Blvd, CB 7426, Chapel Hill, NC 27599-7426 USA

**Keywords:** Children, Diet, Snack, Snacking, Parenting, Schemas, Qualitative

## Abstract

**Background:**

Snacking contributes to excessive energy intakes in children. Yet factors shaping child snacking are virtually unstudied. This study examines food parenting practices specific to child snacking among low-income caregivers.

**Methods:**

Semi-structured interviews were conducted in English or Spanish with 60 low-income caregivers of preschool-aged children (18 non-Hispanic white, 22 African American/Black, 20 Hispanic; 92 % mothers). A structured interview guide was used to solicit caregivers’ definitions of snacking and strategies they use to decide what, when and how much snack their child eats. Interviews were audio-recorded, transcribed verbatim and analyzed using an iterative theory-based and grounded approach. A conceptual model of food parenting specific to child snacking was developed to summarize the findings and inform future research.

**Results:**

Caregivers’ descriptions of food parenting practices specific to child snacking were consistent with previous models of food parenting developed based on expert opinion [1, 2]. A few noteworthy differences however emerged. More than half of participants mentioned permissive feeding approaches (e.g., my child is the boss when it comes to snacks). As a result, permissive feeding was included as a higher order feeding dimension in the resulting model. In addition, a number of novel feeding approaches specific to child snacking emerged including child-centered provision of snacks (i.e., responding to a child’s hunger cues when making decisions about snacks), parent unilateral decision making (i.e., making decisions about a child’s snacks without any input from the child), and excessive monitoring of snacks (i.e., monitoring all snacks provided to and consumed by the child). The resulting conceptual model includes four higher order feeding dimensions including autonomy support, coercive control, structure and permissiveness and 20 sub-dimensions. Conclusions: This study formulates a language around food parenting practices specific to child snacking, identifies dominant constructs, and proposes a conceptual framework to guide future research.

## Introduction

Behavioral aspects of energy imbalance that lead to obesity in young children are multi-factorial, but remain poorly characterized. Snacking has been identified as a potential contributor to excessive energy intakes because children in the United States (US) snack more frequently and consume greater energy from snacks than in past decades [3].

The contribution of snacks to daily energy intake is not trivial; US preschoolers consume approximately 27 % of their daily energy from snacks [3]. Similar patterns have been observed in Chinese [4], Brazilian [5], and British [6] children. Snacking also represents a key source of “empty” calories, which offer few nutrients beyond energy and are seen as the root cause of dietary imbalances [7]. Desserts, salty snacks, and sweetened beverages are top snacks consumed by US children and represent foods high in solid fats and added sugars (SoFAS) [8]. Surprisingly, factors that shape young children’s snacking are virtually unstudied.

Young children consume the majority of their daily energy intake at home [9]. Parents socialize children’s eating behaviors through the types and amounts of foods made available to children in and outside the home, the dietary behaviors they model, and through food parenting styles and practices [10]. While a burgeoning body of research documents the importance of parental feeding styles and practices for shaping children’s obesity risk [11–15], parenting influences on children’s snacking habits have not been appreciably studied. Identifying parents’ feeding goals and practices specific to child snacking is central to understanding parenting influences on snacking. While a number of existing questionnaires include individual items about snacking [16–18], few have been developed to include empirically-based constructs that reflect parenting approaches to feeding children snacks. Moreover, the general literature on food parenting has focused on the negative effects of highly controlling feeding practices such as restriction [19, 20] and pressure to eat [21] to a greater extent than potentially supportive practices. As such, there is a particular need to consider a wider range of parental feeding goals and practices that may be supportive of healthy snacking behaviors in children.

The goal of this study is to advance research on food parenting by qualitatively characterizing practices that caregivers use when feeding snacks to their preschool-aged children. We focus specifically on low-income caregivers given that children in low-income families demonstrate a disproportionate risk of obesity and poor diet quality compared with children from more affluent families [23, 24]. In addition, prior work by members of our research team suggests that low-income mothers of preschool-aged children may perceive snacks as serving a more important role in managing child behavior than in nutrition [25]. Therefore, based on in-depth interviews with low-income parents from diverse racial/ethnic backgrounds, this study will (1) identify food parenting practices specific to snacking, (2) link such practices with higher order food parenting constructs, and (3) develop a conceptual model of food parenting specific to snacking to guide and frame future research including quantitative measures of parenting around child snacking.

## Methods

### Participants

Participants included 60 low-income parents or primary caregivers (18 White, 22 African American, 20 Hispanic) who reported primary responsibility for feeding the target child, aged 3 to 5 years. Caregivers were recruited in Philadelphia and Boston using flyers posted in offices of the Special Supplemental Nutrition Program for Women, Infants and Children (WIC) and online community listings such as craigslist. All caregivers reported eligibility for federal assistance programs such as WIC, Head Start or the Free and Reduced Cost School Meals program. Such programs have income eligibility criteria typically ranging from 100 to 185 % of the federal poverty line. Exclusion criteria included a caregiver younger than 18 years or a  a child with severe food allergy, chronic medical condition or developmental disorder that influenced feeding. All study procedures were reviewed and approved by the Institutional Review Boards at Temple University and Harvard T.H. Chan School of Public Health and all participants completed an informed consent form. Caregivers were compensated with a $45 gift card for their time.

### Design

The current study was part of a larger mixed methods study to delineate parents’ conceptualization of child snacking or their child snacking schemas. Schemas are cognitive frameworks that organize information around concepts [26] and define what might be expected in given situations [27]. Cognitive schemas are often assessed using a card sorting procedure [28, 29]. This article focuses on the semi-structured interview questions which were integrated into a card sorting protocol in which parents were asked to sort a set of 65 cards of snack foods into piles reflecting snacking purposes and contexts. Results from the card sorting procedure are not reported in this study.

### Procedures

Caregivers completed a 60–90 min interview (card sort + interview) in English or Spanish with a trained research assistant. Interviews were conducted at university-affiliated research centers or at a community location close to the participant’s home. An expert in qualitative methods trained the interviewers during a 2 day workshop in qualitative methods including card sort procedures and semi-structured interviews. A semi-structured interview guide with scripted procedures was utilized by all interviewers. Interview questions focused on (a) caregivers’ definitions of snacking, (b) how caregivers decide what, when and how much snack their child eats, and (c) how caregivers respond when their child pesters or nags for a snack (see Table 1). Prior to completing the interview, caregivers completed a number of brief questionnaires assessing demographic characteristics and food security [30].

**Table 1:** Interview guide

1.	When I say the word “snack” what do you think of?
2.	Tell me about your child’s snack habits?
3.	So thinking about [child’s name], why does s/he get snacks?
4.	How do you decide what [child’s name] eats for a snack?
	i. What role does [child’s name] play in this decision?
	ii. Are there snacks that you like [child’s name] to eat? What things do you try to make sure s/he eats those kinds of snacks?
	iii. Are there snacks that you think [child’s name] should eat less often? If yes, what things do you try to do to make sure s/he doesn’t eat too many of those snacks?
	iv. Are there any snacks you particularly enjoy giving [child’s name]? Why is that? When do you tend to offer these kinds of snacks?
5.	How do you decide how much [child’s name] eats for a snack?
	i. What role does [child’s name] have in this decision?
	ii. What things do you do to make sure your child does not eat too much of a particular snack?
6.	How do you decide when [child’s name] eats a snack?
	i. What role does [child’s name] have in this decision?
	ii. When does [child’s name] eat snacks on weekdays? Is this usually about the same time each day?
	iii. How about weekends? Is it usually about the same time each day?
	iv. Tell me about your child’s snack habits between dinner and bedtime?
7.	How do you respond when [child’s name] pesters or nags you for snacks?

### Data analysis

All interviews were audiotaped, transcribed verbatim and later verified by the interviewer. Field notes were completed immediately after each interview to provide a description of the setting and other observations not captured directly through the interview to facilitate data analysis. NVivo10 was used to analyze the transcribed text. An iterative coding process was adopted which included both theory-based and grounded coding [31–33] (see Table 2).

### Code development

A preliminary code list was developed based on existing parenting [34] and food parenting [2, 35] literatures as well as emerging conceptual/theoretical frameworks regarding food parenting, particularly Vaughn’s conceptual map of food parenting [1]. Developed by a team of experts in food parenting, the conceptual map outlines three higher order feeding constructs including coercive control, structure and autonomy support and associated sub-dimensions; for example, sub-dimensions of autonomy support include nutrition education, child involvement, encouragement and support, praise, reasoning and negotiation. Based on this map, a list of anticipated snacking-related food parenting practices under the dimensions of structure, coercive control and autonomy support [36] was created. Three senior authors (KD, CEB, and JOF) with expertise in food parenting coded five randomly selected transcripts using the preliminary code list. This process led to two key modifications. First, food parenting practices reflecting permissiveness (e.g., reluctance to say no to child requests for snacks) were added to the coding scheme given repeated examples of permissiveness identified in the transcripts. Second, coding was broken into a two-step process, as summarized in Table 2, including an initial round of coding with a simplified code list and a second round of coding with a more detailed set of codes to permit theme refinement.

A comprehensive coding manual with standardized operational definitions for each construct was created for each stage of coding. In addition to defining what the specific food parenting practice encompassed, operational definitions included examples of what the construct did not include or how it separated itself from other food parenting constructs. For example, the definition for *Emotion-based feeding of snacks* included the statement “This construct does not include providing food in response to child negative emotions (such as whining or nagging); in this manner it distinguishes itself from providing snacks for behavior management”. The manual was iteratively revised to resolve discrepancies that emerged through inductive analysis and to integrate language and examples from the interviews previously coded. In cases where a code was conceptually modified or added, the coders went back and recoded the data using the revised guide.

Stage one: Initial coding (column 1, Table 2). During stage one coding, food parenting practices were coded without reference to the anticipated parenting dimension(s) to which they were linked. A food parenting practice may reflect multiple parenting dimensions (i.e., autonomy support, coercive control, structure, permissiveness) depending on the valence of the practice. For example, very low limit setting may reflect permissiveness, whereas moderate limit setting might reflect autonomy support, and high limit setting could reflect coercive control. During stage one coding, all examples of a particular food parenting practice were coded as a single unidimensional construct regardless of magnitude or valence (i.e., low, moderate, high). In addition, food parenting practices with considerable overlap (e.g., healthy snacks are available and healthy snacks are accessible) were coded as a single theme. This resulted in identification of 17 food parenting constructs specific to child snacking.

**Table 2:** Summary of data analysis process by which the final constructs were identified

**#**	Stage 1: Constructs initially coded based on theory and existing research	Stage 2: Constructs identified through sub-coding of multidimensional constructs	Stage 3: Final construct name	Anticipated parenting dimension
1	Praise and encouragement of healthy snacks		Praise/encouragement of healthy snacks	Autonomy
2	Child-centered snack provision		Child-centered snack provision	Autonomy
3	Snacks used to build independence and nutrition knowledge		Reasoning and support for healthy snacks	Autonomy
4	Role modeling healthy snacking		Role modeling healthy snacking	Autonomy
5	Snack planning and routines		Snack planning and routines	Structure
6	Prevention or anticipation of child hunger		Snack planning and routines	Structure
7	Snacks to reward behavior		Snacks to reward behavior	Control
8	Snacks to stop nagging and prevent tantrums		Snacks to manage child behavior	Control
9	Pressure to eat snacks		Pressure to eat snacks	Control
10	Snacks to occupy child		Snacks to occupy child	Control
11	Emotion-based feeding of snacks		Emotion-based feeding of snacks	Permissiveness
12	Availability/accessibility of unhealthy snacks	12a. Unhealthy snacks not available or accessible	Availability of healthy snacks	Structure
		12b. Unhealthy snacks are available but not accessible	Restriction of snacks	Control
13	Availability/accessibility of healthy snacks	13a. Healthy snacks are available	Availability of healthy snacks	Structure
		13b. Healthy snacks are accessible	Accessibility of healthy snacks	Structure
14	Snack rules and limits	14a. Excessive rules and limits	Unilateral decision making about snacks	Control
		14b. Moderate rules and limits	Moderate snack rules and limits	Structure
		14c. Absence of rules and limits	No snack rules or limits	Permissiveness
15	Monitoring child snacks	15a. Excessive monitoring of snacks	Excessive monitoring of snacks	Control
		15b. Moderate monitoring of snacks	Monitoring and awareness of snacks	Structure
		15c. Absence of(ambivalence to) monitoring	No involvement with child snacks	Permissiveness
16	Reasoning with child about healthy snacks	16a. Moderate levels of reasoning	Reasoning and support for healthy snacks	Autonomy
		16b. Psychological control through reasoning	Restriction of snacks	Control
17	Responsive to child preferences and demands about snacks	17a. Too responsive to child preferences and demands	No snack rules or limits	Permissiveness
		17b. Overly sensitive to physical and social context of snacking	Context driven provision of snacks	Permissiveness
		17c. Moderately responsive to child preferences and demands	Child-centered provision of snacks	Autonomy
		17d. Not responsive to child snack preferences and demands	Unilateral decision making about snacks	Control

Stage two: Theme refinement (column 2, Table 2). During stage two, coding results from stage one were reviewed to identify food parenting practices that contained more than one theme or parenting dimension. In such cases, sub-coding was performed. Two authors discussed emergent sub-themes that arose from initial coding. The coding guide was modified to incorporate the subthemes. One author (RB) then re-coded the transcripts using the modified guide. Of note, only food parenting constructs that were multidimensional or multi-thematic were re-coded in this manner. As shown in column 2, Table 2 (numbers 12–17), a total of six constructs were sub-coded during stage two. For example, all text coded as *Monitoring child snacks* (#15) was sub-coded as: (15a) *Excessive monitoring*; (15b) *Moderate monitoring*; or (15c) *Absence, or ambivalence to, monitoring*.

Stage three: Construct labeling (column 3, Table 2). In the final coding stage, labels were given to the final list of constructs that could be parsimoniously used in the conceptual model and a parent-report survey currently under development (see column 3, Table 2). Where necessary, constructs with significant conceptual overlap were consolidated. For example, *Absence of snack rules or limits* (14c) and *Too responsive to child preferences and demands* (17a) were both labeled as *No snack rules or limits*.

### Conceptual model development

**Fig.1:**
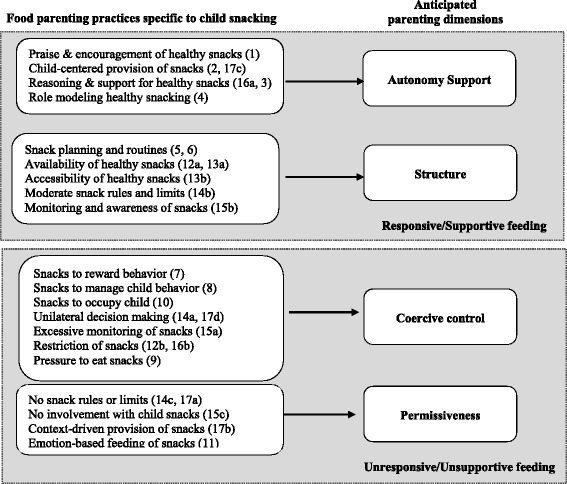
Conceptual model of parents’ food parenting practices specific to child snacking

The final food parenting practices were linked with the original parenting dimensions from which they were derived based on a plausible theoretical association, as agreed upon by author consensus (KD, JOF, CEB, AV) (see column 4, Table 2). The resulting information formed the basis of the final conceptual model for food parenting practices specific to child snacking (Fig. 1). Operational definitions of each construct were generated using an abbreviated version of the definitions from the coding guide and example quotes were identified.

Racial/ethnic differences in food parenting practices specific to child snacking were examined using the matrix function within NVivo which tallies the number of participants who mention a specific food parenting practice and the total number of references to that practice across participants. Given that few noteworthy differences in the tallied frequencies were observed for non-Hispanic white, Black/African American, and Hispanic caregivers, results are presented for the total sample and not by race/ethnicity.

## Results

### Participant characteristics

Caregivers were predominantly mothers, had an average age of 31.2 (8.4) years, and were from diverse racial/ethnic, educational, marital, and employment backgrounds (see Table 3). Half of participating caregivers were obese based on self-reported height and weight and most were enrolled in a federal nutrition program (e.g., WIC, Food stamps).

**Table 3:** Sample characteristics (*N* = 60)

Characteristic	Number	Percent
Sex (female)	56	93.3
Relationship to child		
Mother	55	91.7
Father	3	5.0
Other caregivers	2	3.3
Race/ethnicity		
White	17	28.3
Black/African American	23	38.3
Hispanic/Latinoa(a)	20	33.3
Primary languge		
English only or primariliy	45	75
English and Spanish	3	5
Spanish only or primarily	12	20
Educational background		
Less than high school	10	16.6
High school graduate/GED	18	30.0
Technical school/some college	23	38.3
College graduate or higher	9	15.0
Employment status		
Employed	22	36.7
Unemployed	28	46.7
Other or missing	10	16.6
Full time student (yes)	20	33.3
Marital status		
Married or living with partner	23	38.3
Divorced/separated	5	8.3
Single	32	53.3
Weight status		
Underweight	2	3.3
Normal weight	17	28.3
Overweight	11	18.3
Obese	30	50.0
Experienced food insecurity in past year (yes)	26	43.3
Family enrollment in federal assistance programs		
WIC	42	70.0
Food stamps/SNAP	48	80.0
Free/reduced cost school meals	28	46.7
Head Start	21	35.0

*GED* General Educational Development (high school completion certificate); *WIC* Special Supplemental Nutrition Program for Women, Infants and Children; *SNAP* Supplemental Nutrition Assistance Program

### Conceptual model

The resulting conceptual model, presented in Fig. 1, summarizes the final list of food parenting practices organized by their anticipated higher order parenting dimension. Autonomy support and permissiveness each included four food parenting practices. Structure included five and coercive control included seven food parenting practices. Table 4 lists the operational definitions for each food parenting practice along with (a) the number of caregivers (i.e., number of sources) who mentioned a particular food parenting practice and (b) the number of times a practice was mentioned across all interviews (i.e., number of references). Illustrative quotes for each food parenting practice, organized by parenting dimension, are summarized below.

**Table 4:** Food parenting practices specific to child snacking and their operational definitions

Parenting dimensions and snacking-related food parenting practice	Operational Definition	# of caregivers^1^	# of references^1^
AUTONOMY SUPPORT		47	126
Praise/encouragement of healthy snacks	Uses verbal praise and encouragement to reinforce healthy snacking behaviors.	5	5
Child-centered provision of snacks	Responsive to the child’s hunger when making decisions about the child’s snacking needs including food preferences and amount consumed. Prompts child to assess hunger/fullness cues.	22	39
Reasoning and support for healthy snacks	Provides physical assistance, explanations, and reasoning to facilitate child learning and/or independence around snacking.	46	108
Role modeling healthy snacking	Intentionally uses own healthy snacking behaviors/choices as a guide for the child.	17	17
STRUCTURE		54	280
Snack planning and routines	Plans snack foods and timing which results in a consistency and predictability in the context of snacking.	49	168
Availability of healthy snacks	Ensures child receives healthy snacks by keeping healthy foods in the home and making them available at snack time. Also includes limiting availability of unhealthy snacks by keeping them out of the home and limiting impulse snack purchases while out with child.	32	69
Accessibility of healthy snacks	Facilitates child’s access to and consumption of healthy snacks through physical availability (e.g. keeping healthy foods in places child can see and easily access) and appealing preparation (e.g. using prepackaged healthy foods, tasty dips for fruit or vegetables).	27	50
Moderate snack rules and limits	Setting reasonable or moderate limits around what, when, how much of snacks are offered to children through guided choices, reasonable rules, or modifications to a child’s requests or preferences. Examples include not allowing snacks too close to dinner (reasonable rule) and offering water instead of soda, or 2 cookies instead of 5 as requested by the child (modifications to child requests).	19	40
Monitoring and awareness of snacks	Keeps track of child’s snack intake in a developmentally appropriate manner by keeping track of the timing, portion size, and type of snacks consumed.	22	40
Parenting dimensions and snacking-related food parenting practice	Operational Definition	# of caregivers^1^	# of references^1^
COERCIVE CONTROL		54	278
Snacks to reward behavior	Provides snacks to reward the child for desired behaviors (e.g., eats their dinner, follows directions/routine, good behavior or grades in school).	37	131
Snacks to manage child behavior	Reactive strategies whereby parent provides a snack to interrupt a negative behavior (e.g., nagging) or to pre-empt the escalation of the behavior (e.g. tantrum).	26	63
Snacks to occupy child	Proactive strategies or actions in which snacks are used to keep the child quiet or to distract or otherwise occupy the child in contexts where disruptive behavior is not acceptable (e.g. car, church, when parent is occupied).	19	40
Unilateral decision making about snacks	Decides in a unilateral manner if, when, and how much their child may have for a snack without regard for their child’s preferences or previous intake in a given day. Child is told to accept what parent offers or have nothing at all.	17	34
Excessive monitoring of snacks	Goes to great lengths to monitor everything the child eats for a snack in order to control consumption (type, portion size, and timing). Concern and awareness of child’s snack is expressed to the child and other caregivers. This does not include developmentally appropriate surveillance of child’s snacks expected for the age of the child (see “Monitoring and awareness of snacks”).	4	6
Restriction of snacks	Utilizes rigid emotional and physical strategies to limit child’s access to and intake of unhealthy foods. These strategies may include emotional coercion (e.g. threatening sickness or punishment for eating candy), excessive rule setting (e.g. child is never allowed to consume candy), or overt punishment for consuming a prohibited food. Physical strategies include keeping foods present, but out of the child’s reach (e.g. using locks to restrict child access to snack cabinet), and physically taking snacks away from the child.	19	28
Pressure to eat snacks	Encourages child to increase intake of a particular snack using strategies that disregard the child’s preferences or requests through verbal prompts (e.g. pleading), sitting and watching child (e.g. observing every bite), or threatening punishment if food is not eaten.	11	19
Parenting dimensions and snacking-related food parenting practice	Operational Definition	# of caregivers^1^	# of references^1^
PERMISSIVENESS		37	115
No snack rules or limits	Places few to no limits on what, when and how much of a snack a child consumes. Unhealthy snacks may be readily available to child without limits. Parent may still have awareness of what snacks child is eating (see “No involvement” below), but not feel they have control over child’s choices.	24	41
No involvement with child snacks	Lacks awareness of child’s daily snack consumption and is uninvolved with the child’s regulation of intake. This construct is distinct from “No rules about snacks” in that parents are completely disengaged from what child is eating.	15	31
Context-driven provision of snacks	Allows child’s snack consumption to be influenced by external pressures related to the social environment (e.g. pressure from grandparent) or context of eating occasion (e.g. always gets an ice cream if the truck drives by). Parent does not act as a buffer between the child and the social environment.	19	26
Emotion-based feeding of snacks	Uses snacks to show the child they love him/her or to make the child happy.	17	31

^1^The column totals may not be equal to the sum of the categories making up that column in instances where a text passage was double coded as reflecting more than one construct. In such cases, the passage would only be counted once toward the total number of references for the associated parenting dimension. A similar approach was used to calculate the total number of caregivers (i.e., a caregiver was only counted once for each parenting dimension although s/he may have provided multiple examples of that construct)

### Autonomy support

Forty seven caregivers mentioned at least one of the food parenting practices reflecting autonomy, with a total of 126 references to these practices across all caregivers. The most frequently mentioned practice was *Reasoning or support*, which was defined as using snacks as an opportunity to promote independence and build child nutrition knowledge. A total of 46 caregivers mentioned this food parenting practice at least once, with 108 references to it across all interviews. Caregivers recalled strategies such as having the child assist with preparing a snack or describing how a particular snack may affect the child's health or energy levels. *Child-centered provision of snacks* and *Role modeling of healthy snacking* were mentioned to a moderate degree across caregivers with 22 and 17 caregivers mentioning each approach respectively. Surprisingly few examples *of Praise and encouragement* were identified. Examples of the three predominant food parenting practices specific to snacking that reflect autonomy support include the following:

*Child-centered snack provision: If he’s hungry and it’s after school or it’s in between meals, he asks and tells me he’s hungry. I’ll always give him something to eat if he says that he’s hungry. (White mother of 4-year-old boy)*

*Reasoning and support for healthy snacks: The peanut butter and celery. . . I think it’s fun to make like she can help me spread the peanut butter on there and she enjoys eating it and I know it’s good for her. Um, sometimes we make like the fruit kabobs like where we’ll dice the fruit. So I’m trying to work on cutting with her too. (White mother of 4-year-old girl)*

*Role modeling healthy snacking:* I'm trying to get her to eat broccoli. . . Like, I was started to sit down with her like, “Look, this is good, Mami. Hey look at Mommy eating it.” (Hispanic mother of a 4-year-old girl)

### Structure

Fifty four caregivers mentioned a snacking-related food parenting practice that reflected structure, with a total of 280 references to such practices identified across all caregivers. The most commonly recalled approach was *Snack planning and routines* with 168 references identified. *Availability and accessibility of healthy snacks*, *Moderate snack rules and limits* and *Monitoring and awareness of snacks* were also mentioned at least moderately with 40 references each. Examples of each food parenting practice reflecting structure include the following:

*Snack planning and routines:* Like I got-I got ‘em on a schedule so he know that he gonna get a snack at this time. (African American father of a 3-year-old boy)

*Availability of healthy snacks:* I don’t keep chips in the house, so if we was in the house, she would grab-I keep the fruit snacks, fruit cups and apple sauce inside the house, so she would grab this more when she in the house. (African American mother of a 3-year-old girl)

*Accessibility of healthy snacks:* I try to dice him oranges, or buy the apples in the bags. That’s easier for him to grab versus – of course, he can't prepare that his self. So if there’s something there fast as the bag of chips, you can go in the refrigerator and get a Ziploc bag of oranges that I cut up, or you know the Motts Apples-to-Go or apple sauce. (African American mother of a 5-year-old boy)

*Moderate snack rules and limits:* He wanted Oreos. You know, he had the whole pack ready to eat them. I gave him two, and that was it, so he was happy with the two. Now if I didn’t see him, he would have went through the whole box. I’ll, you know, I’ll compromise. If he wants some, he’s not going to have ten of them but I give him two. (White mother of a 3-year-old boy)

*Monitoring and awareness of snacks:* If I know she ate a lot or she ate a big meal, then my snack will be limited because I know how could you have room for anything else if you just ate? You know, let the food digest. (African American mother of a 4-year-old girl)

### Coercive control

Snacking-related food parenting practices reflecting coercive control were also frequently mentioned with 278 references from 54 caregivers. The most frequently mentioned approaches include using *Snacks to reward behavior* and *Snacks to manage behavior* with approximately half of caregivers mentioning such approaches. *Using snacks to occupy a child*, *Restriction of snacks*, *Unilateral decision making about snacks* and *Pressure to eat snacks* were mentioned by approximately 20–30 % of caregivers. Very few caregivers reported *Excessive monitoring of snacks*. Some examples of coercive food parenting practices include the following:

*Snacks to reward behavior:* Because, like I said, I buy cheeseburgers at McDonalds, that’s my reward for them behaving at church. (Hispanic mother of a 4-year-old girl).

*Snacks to manage behavior:* He just wanna constantly, “I’m hungry. I’m hungry. I’m hungry,” so I turned around and give him a bag of chips or something or, like, a piece of candy or something. “Boy, just be quiet and wait until I’m finished. It’s almost done.” (African American mother of a 5-year-old boy)

*Restriction of snacks:* You don’t open those cabinets unless you ask me first or you will be punished immediately. Um, the same thing with opening the fridge. You know you don’t get to touch any of those things. You know you didn’t pay for it, you don’t know whose it is; don’t touch it. (White mother of a 5-year-old girl)

*Unilateral decision making about snacks:* Um, I give it to him with no choice, like he has to eat these or he eats nothing at all. He gets no snack. (African American mother of a 3-year-old girl)

### Permissiveness

Thirty seven, or slightly more than half of caregivers, made a total of 115 references to using permissive food parenting practices in the context of snacking. Such practices – which included *No snack rules or limits*, *No involvement in child snacking*, *Context-driven provision of snacks*, and *Emotion-based feeding of snacks* – were each reported between 25 and 41 times across 37 caregivers. Examples of food parenting practices reflecting permissiveness include the following:

*No snack rules or limits:* Oh, he’s eating throughout the day. I mean, it’s not like only certain times or anything. It’s like when he wants a snack, I’ll give him a snack. I don’t ever want to deny him something, you know, be mean to him, be the hateful father. (White father of a 3-year-old boy)

*No involvement with snacks:* I never know who’s really feeding him. (African American mother of a 5-year-old boy)

*Context-driven provision of snacks:* Or she sees somebody eating something. She’ll be like, “Mom.” So, say the ice cream truck – buy her ice cream, or ice cream pops. There’s like a corner store. She’ll tell me she wanna go. I’ll take her and she’ll get chips and juice. Or, donuts, or she likes some Tasty Cakes. (Hispanic mother of a 3-year-old girl)

*Emotion-based feeding of snacks:* To, make him feel better I gave him ice cream. So it was ice cream and candy. It – it helped, you know. He was crying, but then the tears went away. (White father of a 3-year-old boy)

## Discussion

This qualitative study examined food parenting practices specific to child snacking among low-income non-Hispanic white, African American and Hispanic caregivers who were predominantly mothers. While a recent Delphi study compiled the opinions of research experts regarding anticipated food parenting practices used in the context of snacking [2], this is the first comprehensive assessment of such practices from the perspective of caregivers. Given that snacks consumed by children are often calorie-dense and high in SoFAS [7], greater understanding of the approaches parents use to feed their children snacks may highlight important intervention targets. While the provision of snacks to young children is generally encouraged [37] there is virtually no information to guide parents’ decision-making about when or under what circumstances it is appropriate or recommended to provide a snack. This study serves as a precursor to the assessment of these important areas for research by developing a language around food parenting specific to child snacking and identifying predominant constructs that should be assessed further in future work.

Parents’ descriptions of their snacking-related food parenting practices were consistent with what has been previously observed for food parenting practices in general [1, 34, 35, 38] and specific to child snacking as reported in the Delphi study [2]. There were a few exceptions, however. First, in this study permissive feeding approaches were mentioned by more than half of the caregivers. As such, they warranted greater consideration as a food parenting strategy in the context of snacking than has previously been considered. While permissive feeding styles have been discussed in reference to child obesity [39, 40], less attention has been directed toward the specific parenting practices that reflect permissive feeding approaches. We propose that permissive feeding should be conceptualized as a higher order construct with multiple sub-constructs when child snacking is the focal context. A stronger emphasis on permissive approaches to providing children snacks compared with a general feeding context not specific to snacking makes sense given that snacks, particularly “tasty snacks” or “treats”, are often the vehicle through which permissive practices such as emotion-based feeding and feeding as a reward are expressed.

A second difference is that our model does not include the construct of food preparation (i.e., the methods parents employ to prepare foods which can affect their healthfulness), which is included in Vaughn’s concept map. Our qualitative findings do not support the relevance of food preparation for child snacking. Similarly, food parenting experts did not identify food preparation as a pertinent construct in the Delphi study of snacking-related food parenting [2]. A third difference is that a number of practices delineated by Vaughn [1] (i.e., nutrition education, child involvement and reasoning) were better captured in this study as a single practice when related to snacking. Finally, our model presents a number of novel food parenting practices not included in prior models [1, 2] including child-centered feeding of snacks, unilateral decision making and excessive monitoring of snacks. While we have explicitly compared our model with existing models to ensure that readers can integrate information across studies, it is worth noting that all models outlined are hypothetical at this point and have not been explicitly tested.

The primary strength of this study is the detailed assessment of parents’ approaches to feeding children snacks. While there is accumulating evidence that children’s snacking behaviors may place them at risk of obesity and it is widely recognized that parents are highly influential in shaping children’s diet behaviors, food parenting practices specific to snacking have received very little attention. Additional strengths of the study include the compilation of a relatively large number of in-depth interviews with low-income parents from racial/ethnically diverse backgrounds and the utilization of multiple theoretical models. Noted strengths of the study need to be weighed against study limitations including the inability to test or validate the model proposed and the low number of fathers who participated. While fathers were eligible to participate, they were not explicitly targeted which may be necessary to successfully engage fathers in such research. Despite these weaknesses, this study clearly informs future research by articulating dominant constructs in food parenting specific to child snacking which warrant greater consideration. It also presents a conceptual model that can be used to frame and focus future research and which could be iteratively updated and revised based on the data gathered.
